# Stress, anxiety and depression in clinical nurses in Vietnam: a cross-sectional survey and cluster analysis

**DOI:** 10.1186/s13033-018-0257-4

**Published:** 2019-01-03

**Authors:** Thi Thu Thuy Tran, Ngoc Bich Nguyen, Mai Anh Luong, Thi Hai Anh Bui, Thi Dung Phan, Van Oanh Tran, Thi Huyen Ngo, Harry Minas, Thuy Quynh Nguyen

**Affiliations:** 1 Faculty of Environmental and Occupational Health, Hanoi University of Public Health, 1A Duc Thang Road, Duc Thang Ward, North Tu Liem District, Hanoi, Vietnam; 2 Health and Environment Management Agency, Ministry of Health, Line 8, Ton That Thuyet Street, My Dinh 2, Nam Tu Liem District, Hanoi, Vietnam; 3 National Institute of Hygiene and Epidemiology, 1 Yecxanh Street, Hai Ba Trung District, Hanoi, Vietnam; 4 Nursing Office, Viet Duc University Hospital, 40 Trang Thi Street, Hanoi, Vietnam; 5 Global and Cultural Mental Health Unit, Centre for Mental Health, Melbourne School of Population and Global Health, 235 Bouverie Street, Carlton, VIC 3053 Australia

**Keywords:** Stress, Anxiety, Depression, Clinical nurses, Cluster analysis, Workload, Work relationship

## Abstract

**Background:**

Hospital nurses are exposed to various work-related factors that may be associated with increased risk of developing different mental disorders. Empirical evidence on the prevalence and correlates of individual mental health problems such as stress, anxiety and depression is widely reported, while a combined pattern of these conditions is unknown. This study aims to examine the co-occurrence of stress, anxiety and depression among clinical nurses, and to explore socio-demographic characteristics of, and working conditions experienced by, nurses that may be associated with these three mental health conditions.

**Methods:**

A cross-sectional study was implemented in one tertiary hospital in Hanoi city, Vietnam, from May to September 2015. A self-reported questionnaire including a short version of the Depression, Anxiety and Stress scale 21 items and questions on demographic and work-related characteristics was delivered to 787 registered nurses. 600 completed questionnaires was used in the final analysis (76.2% response rate). The two-step clustering analysis was performed to identify sub groups. Chi square test and post hoc ANOVA analysis with Bonferroni correction were used to examine differences in psychological status, demographic characteristics and working conditions among the clusters (two-tailed p < 0.05).

**Results:**

The prevalence of self-reported stress, anxiety and depression were 18.5%, 39.8% and 13.2%, respectively. 45.3% participants reported symptoms of at least one mental disorder, 7.3% had all three. Nurses in the first cluster (high prevalence of mental disorders), had high task demand and conflict at work with low job control and reward. The second cluster nurses (moderate percentage of mental strain) were significantly older and in marital relationship, high task demand and job control, and presence of chronic diseases. The lowest proportion of self-perceived mental disorders were observed in the cluster three who were younger and had fewer years of services, moderate task demand and low job control and better physical health in comparison with those in the other two clusters (p < 0.05).

**Conclusions:**

Stress, anxiety and depression were prevalent among clinical nurses. Heterogeneity in demographic characteristics and working conditions were observed across clusters with different patterns of mental disorders. Institutional effort should be emphasized to support nurses in their career development to reduce psychological strains.

**Electronic supplementary material:**

The online version of this article (10.1186/s13033-018-0257-4) contains supplementary material, which is available to authorized users.

## Background

In recent years, nursing has been reported to be one of the most stressful professions in both developed and developing countries [1–4]. Nurses appear to suffer more severe mental health problems than other health practitioners in clinical positions [5] and the general population [6]. Among these disorders, depression, anxiety and stress are the most prevalent and have received the most attention in psychological research among nurses [7, 8].

The individual and organizational impacts of stress, anxiety and depression are widely documented. Mental disorders are significantly associated with absence from work, intention to leave, and high turnover [9, 10]. The presence of one or more of these mental health problems can contribute to occupational accidents [11, 12], impaired work performance and errors of judgment, and a negative attitude at work [13]. Moreover, nurses’ mental health problems could endanger the lives and satisfaction of hospital patients and the quality of services provided [14], and for their employing organization can contribute to reputational damage, and reduced productivity and clinical effectiveness [15].

Factors contributing to elevated levels of stress among health care workers could be described as work-related and non-work-related factors. Several conditions of work are associated with higher risk of psychological stress, including insecure employment status, heavy workload, emotional response to suffering and dying patients, organizational problems and conflict, and workplace violence [16–19]. Among personal characteristics, age, marital status [20, 21], and self-perceived health status [2, 22, 23] have been reported to be important in epidemiological studies.

While the symptomatology of mental disorders is complex, and common mental disorders such as depression, anxiety and stress are known to commonly co-occur, interactions between these disorders are unclear, particularly among nurses. Most studies have explored only single mental disorders [7, 12, 16, 18, 20, 24–28], or have reported findings on stress, anxiety and depression separately, even in studies that have measured all three [6, 23]. However, it would be potentially useful to concurrently measure and interpret anxiety, depression and stress as a whole in order to comprehensively describe the mental health status of study subjects and to prevent unnecessary duplication of research and intervention efforts [29]. A useful, validated [30, 31] self-report instrument for the concurrent measurement of depression, anxiety and stress is the Depression Anxiety Stress Scales, available in long (DASS-42) and short versions (DASS-21) [32]. It has also been suggested that the DASS scale is particularly sensitive in discriminating anxiety from depression [29].

In Vietnam, mental disorders have been studied in several occupations. However, published studies of the nursing profession have only reported on mental disorders, such as stress, anxiety and depression [5, 6, 33–36]. Meanwhile, Vietnamese provincial hospitals are characterized by stressful working environments, high workload, a shortage of skilled health staff, and inadequate infrastructure and medical equipment [37, 38]. Work factors such as these may be expected to be associated with development among nursing and other staff of multiple types of mental health problems. Hence, the aim of this study was to examine the co-occurrence of stress, anxiety and depression among clinical nurses, and to explore socio-demographic characteristics of, and working conditions experienced by, nurses that may be associated with these three mental health conditions.

## Methods

### Study design, setting and procedure

An institution-based cross-sectional study was implemented in one tertiary hospital in Hanoi city, the capital of Vietnam, from May to September 2015. This was the national hospital specialized in surgery. Most patients were admitted to the hospital with severe conditions due to accidents or on referral from lower level health care facilities. The study sample consisted of registered nurses who had been working in the hospital over 1 year, were participating in clinical care and had an employment contract with the hospital.

A sample size was calculated to estimate a population proportion with specified absolute precision. The sample calculation was performed by WHO sample size software [39] with the anticipated proportion of mental disorders (depression, anxiety or stress) as 0.5 to achieve the largest sample size, an absolute precision of 10% of the true proportion at 95% confidence. Response rates for cross-sectional study in hospital settings greatly varied from 36.7% [27] to 89% [40]. With the expected response rate set at 50%, the required number of nurses was 770. Hence, all of 787 nurses meeting the inclusion criteria were invited to participate in the study.

An explanatory letter concerning the study, a consent form and study questionnaires were sent to 787 nurses. The questionnaires included the self-administrated short form Depression, Anxiety and Stress scale (DASS 21), 10 questions on demographic data and 16 questions on working conditions (See Additional file 1). 621 questionnaires were returned, of which 21 questionnaires had missing data on some questions. Participation was voluntary and confidentiality was guaranteed. Only 600 completed questionnaires were entered into the final database for analysis (response rate 76.2%). The average age of the study sample was 33 (SD = 7.5). A majority of participants were female (77.8%) and in a marital relationship (80.7%). More than half of the participants were over 30 years old (52.3%) and had worked for more than 5 years in the hospital (58.7%).

### Measurements

#### Outcome variables

The study used the self-administrated short form Depression, Anxiety and Stress scale (DASS 21) with three sub-scales: DASS 21-stress, DASS21-anxiety and DASS 21-depression to investigate stress, anxiety and depression within 1 week prior to the survey [32]. Each sub-scale contained 7 questions with Likert response scale ranging from 0 (Did not apply to me at all) to 3 (Applied to me very much or most of the time). Scores for depression, anxiety and stress were calculated by summing the scores for the relevant items of each sub-case then multiplied by two, following the Scale manual [41]. Higher scores indicated higher level of severity in each dimension. The scores were also categorized as “normal”, “mild”, “moderate”, “severe”, and “extremely severe” in each sub-scale (Table 1) [41].

**Table 1:** DASS severity levels

Category	Depression	Anxiety	Stress
Normal	0–9	0–7	0–14
Mild	10–13	8–9	15–18
Moderate	14–20	10–14	19–25
Severe	21–27	15–19	26–33
Extremely severe	28+	20+	34+

The DASS 21 has been translated and validated among Vietnamese women [42] and adolescents [43]. The reliability and validity of DASS 21 among health care and nursing populations had been widely reported [44, 45]. In this study, the Cronbach alphas based on standardized items for the whole scale, DASS 21-stress, DASS21-anxiety and DASS 21-depression were 0.89, 0.78, 0.74 and 0.74, respectively.

A variable of combined mental disorders was computed by adding all three categorical variables of stress, anxiety and depression. The number of disorders ranged from 0 (not any disorder) to 3 (all three disorders). The cut-points for the presences of indicators of stress, anxiety and depression were over 14, 7 and 10 respectively (Table 1) [41].

#### Covariates

Demographic variables included age (3 groups: under 31 years old, from 31 to 35 and above 35 years old), years working for the hospital (3 groups: under 6 years, from 6 to 10 years and above 10 years), gender, marital status (dichotomous response of married and lived with spouse and single/divorced/widowed), education (3 groups: vocational training, college degree and university and higher degree), contribution to family finances (dichotomous response of more than 50% and less than 50%), conditions of some chronic diseases including compensated occupational diseases (such as occupational hepatitis B and C, HIV/AIDS and Tuberculosis), metabolism disorders, musculoskeletal disorders and cardiovascular disorders (dichotomous response of yes and no).

Working conditions contained ward type (dichotomous groups of surgical wards and others), management responsibility (dichotomous response of yes and no), types of employment contract (temporary or permanent), frequency of caring for severe patients and doing tasks out of responsibility (frequently and occasionally or none), perception suitability of the work for a nursing professional, health and income (suitable or not suitable), perception of work pressure (high pressure or normal to low pressure), opportunity for career training (yes or no), relationship with coworkers, supervisors and patients (good or normal/bad), conflict with coworkers and supervisors (yes or never), and intention to work at the hospital in the next 5 years (yes or no).

#### Statistical analysis

Statistically, the most appropriate method to identify data patterns and groups of subjects with similar characteristics is cluster analysis [46]. This method enables the exploration of the substantial heterogeneity in the participants’ characteristics [47]. In this study, two-step clustering analysis using five variables (stress, anxiety and depression scores, age and number of years working in the hospital) was carried out to categorize participants into different groups/clusters. The two-step clustering approach is appropriate when the number of clusters is not known in advance [46, 48]. The choice of similarity measure and number of clusters were based on Bayesian information criterion (BIC) values [49, 50]. After clusters were formed within the sample, group comparison was performed. Descriptive analysis was used to describe the characteristics of the study sample and the status of stress, anxiety and depression. Chi square test and post hoc ANOVA analysis with Bonferroni correction were used to examine any significant differences in psychological status, demographic characteristics and working conditions among the clusters with a significance of p < 0.05 (two-tailed). Data were processed with EpiData 3.0 and analyses were conducted with SPSS v.16 (SPSS Inc., Chicago, IL, USA).

## Results

The two-step cluster analysis yielded three clusters (BIC change = − 301.6). The number of participants in cluster 1 (n = 105), 2 (n = 133) and 3 (n = 362) and the clusters accounted for 17.5%, 22.2% and 60.3% of the whole sample, respectively. The three clusters were formed based on similarity in their responses to questions on stress, anxiety, depression, age and years of services in the hospital.

### Comparison among clusters

#### Demographic characteristics

As shown in Table 2, a majority of participants were: under 36 years old (74%, mean age of the whole sample was 33 ± 7.5), female nurses (77.8%), completing nursing vocational training (65.5%), in a marital relationship and currently living with a spouse (80.7%), responsible for over half of family finances (79.7%) and had worked more than 5 years in this hospital (58.7%). Some nurses reported chronic disease such as compensated occupational diseases (4%), metabolism disorders (7.5%), musculoskeletal disorders (20.3%) or cardiovascular diseases (8.3%).

**Table 2:** Demographic characteristics of the whole sample and three clusters

Demographic characteristics	Whole sample (n = 600)		Cluster						Overall		Between clusters^a^		
			1 (n = 105)		2 (n = 133)		3 (n = 362)				1 to 2	1 to 3	2 to 3
	n	%	N	%	N	%	n	%	χ^2^	P	p	P	p
Age (mean ± SD)	33 ± 7.5		31 ± 4.5		44 ± 6.7		29 ± 3.2				< 0.01	***0.05***	< 0.01
< 31	286	47.7	52	49.5	0	0.0	234	64.6	474.8	*<* *0.01*	*< 0.01*	*< 0.01*	*< 0.01*
31–35	158	26.3	39	37.1	2	1.5	117	32.3					
> 35	156	26.0	14	13.3	131	98.5	11	3.0					
Gender													
Male	133	22.2	26	24.8	22	16.5	85	23.5	3.2	0.20	0.14	0.80	0.11
Female	467	77.8	79	75.2	111	83.5	277	76.5					
Education													
Vocational training	393	65.5	68	64.8	82	61.7	243	67.1	4.9	0.29	0.63	0.16	0.31
College	92	15.3	12	11.4	21	15.8	59	16.3					
University and higher	115	19.2	25	23.8	30	22.6	60	16.6					
Marital status													
Single, divorced, widowed	116	19.3	25	23.8	7	5.3	84	23.2	21.7	*<* *0.01*	*<* *0.01*	0.90	*<* *0.01*
Married lived with spouse	484	80.7	80	76.2	126	94.7	278	76.8					
Family financial contribution													
≥ 50%	478	79.7	91	86.7	120	90.2	267	73.8	20.1	*<* *0.01*	0.42	*< 0.01*	*<* *0.01*
< 50%	122	20.3	14	13.3	13	9.8	95	26.2					
Years at the hospital	9 ± 7.7		6 ± 4.6		21 ± 6.9		5 ± 2.8				*< 0.01*	0.06	*< 0.01*
< 6	248	41.3	47	44.8	0	0.0	201	55.5	459.9	*< 0.01*	*< 0.01*	*< 0.01*	*< 0.01*
6–10	193	32.2	44	41.9	2	1.5	147	40.6					
> 10	159	26.5	14	13.3	131	98.5	14	3.9					
Compensated occupational diseases with success claim and financial support													
Yes	24	4.0	7	6.7	5	3.8	12	3.3	2.4	0.30	0.38	0.16	0.79
No	576	96.0	98	93.3	128	96.2	350	96.7					
Metabolism disorders (obesity, diabetes and dyslipidemia)													
Yes	45	7.5	11	10.5	22	16.5	12	3.3	26.2	*<* *0.01*	0.19	*<* *0.01*	*<* *0.01*
No	555	92.5	94	89.5	111	83.5	350	96.7					
Musculoskeletal disorders													
Yes	122	20.3	21	20.0	59	44.4	42	11.6	64.4	*<* *0.01*	*<* *0.01*	***0.03***	*<* *0.01*
No	478	79.7	84	80.0	74	55.6	320	88.4					
Cardiovascular diseases (Hypertension, hypotension)													
Yes	50	8.3	5	4.8	31	23.3	14	3.9	50.2	*<* *0.01*	*<* *0.01*	0.78	*<* *0.01*
No	550	91.7	100	95.2	102	76.7	348	96.1					

^a^p from Chi square test for percentage comparison and Post-hoc ANOVA for mean comparison (two-tailed)

Italic: < 0.01, bolditalic: < 0.05

Several significant differences in demographic characteristics were presented across the three clusters, particularly age, marital status, family financial contribution, years working at the hospital and chronic diseases. Cluster 2 was characterized by the oldest group (98.5%, mean age 44 ± 6.7), married (94.7%), main support of family finance (90.2%), longest years of services in the hospital (98.5%, mean 21 ± 6.9) and higher percentages of chronic diseases (excluding compensated occupational disease). These features of cluster 2 was substantially different to those of cluster 1 and cluster 3 (mostly at significance level < 0.01). Cluster 3 contained a larger proportion of younger nurses (64.6%, mean age 29 ± 3.2), who had worked the least number of years in the hospital (55.5% under 6 years, mean 5 ± 2.8), and fewer making the major contribution to family finances than in the other two clusters (73.8% versus 86.7% of cluster 1 and 90.2% of cluster 2). Characteristics of participants in cluster 1 were between those in the other two clusters. No significant differences were observed between clusters in gender, education level, and presence of compensated occupational diseases.

#### Status of stress, anxiety and depression

Table 3 shows that the prevalence of indicators of stress, anxiety and depression were 18.5%, 39.8% and 13.2%, respectively. 45.3% of participants had symptoms of at least one mental disorder. The distribution of self-perceived stress, anxiety and depression cases and level of severity were significantly different across the three clusters, with cluster 1 containing the largest proportions of nurses with stress (73.3%), anxiety (86.7%) and depression (61.9%) than subjects in the other two clusters (p < 0.01). Significantly larger proportions of nurses in cluster 1 suffered from severe and extremely severe levels of all three mental disorders in comparison with clusters 2 and 3 (p < 0.01). The mean stress, anxiety and depression scores in cluster 1 were also significantly higher than those of clusters 2 and 3 (p < 0.01). The only difference between cluster 2 and 3 was the percentage of depression cases, which was greater in cluster 2 than in cluster 3 (p < 0.01). Regarding the number of disorders, 100% of nurses in cluster 1 reported at least one psychological disorder and nearly 40% had all three. These figures were significantly higher than those of the other two clusters (p < 0.01). Less than one-third of nurses in cluster 2 and 3 reported symptoms of one disorder (27.8% and 28.8%, respectively). More nurses in cluster 2 suffered from multiple mental disorders than those in cluster 3 (p < 0.05). Only three nurses in cluster 2 (2.2%) had all three problems and no nurses in cluster 3 had all three problems.

**Table 3:** Stress, anxiety and depression in the whole sample and three clusters

Mental disorders	Whole sample (n = 600)		Cluster						Overall		Between clusters^a^		
			1 (n = 105)		2 (n = 133)		3 (n = 362)				1 to 2	1 to 3	2 to 3
	n	%	n	%	n	%	n	%	χ^2^	p	p	p	p
Stress	10.5 ± 6.4		19.2 ± 5.9		9.2 ± 5.6		8.4 ± 4.5				< 0.01	< 0.01	0.29
Normal	489	81.5	28	26.7	120	90.2	341	94.2	227.3	*<* *0.01*	*<* *0.01*	*<* *0.01*	***0.024***
Mild	54	9.0	29	27.6	7	5.3	18	5.0					
Moderate	42	7.0	33	31.4	6	4.5	3	0.8					
Severe	12	2.0	12	11.4	0	0.0	0	0.0					
Extremely severe	3	0.5	3	2.9	0	0.0	0	0.0					
Anxiety	6.7 ± 5.6		13.9 ± 6.9		5.2 ± 4.5		5.1 ± 3.6				*<* *0.01*	*<* *0.01*	1.00
Normal	361	60.2	14	13.3	90	67.7	257	71.0	229.9	*<* *0.01*	*<* *0.01*	*<* *0.01*	0.15
Mild	80	13.3	8	7.6	17	12.8	55	15.2					
Moderate	117	19.5	47	44.8	22	16.5	48	13.3					
Severe	25	4.2	20	19.0	3	2.3	2	0.6					
Extremely severe	17	2.8	16	15.2	1	0.8	0	0.0					
Depression	4.5 ± 4.8		11.4 ± 5.4		3.7 ± 3.8		2.7 ± 2.7				*<* *0.01*	*<* *0.01*	1.00
Normal	521	86.8	40	38.1	125	94.0	356	98.3	269.2	*<* *0.01*	*<* *0.01*	*<* *0.01*	*<* *0.01*
Mild	52	8.7	41	39.0	5	3.8	6	1.7					
Moderate	18	3.0	15	14.3	3	2.3	0	0.0					
Severe	8	1.3	8	7.6	0	0.0	0	0.0					
Extremely severe	1	0.2	1	1.0	0	0.0	0	0.0					
Number of disorders													
Not any	328	54.7	0	0	84	63.2	244	67.4	365.6	*<* *0.01*	*<* *0.01*	*<* *0.01*	***0.02***
1 disorder	159	26.5	18	17.1	37	27.8	104	28.8					
2 disorders	69	11.5	46	43.8	9	6.8	14	3.8					
All 3 disorders	44	7.3	41	39.1	3	2.2	0	0					

^a^p from Chi square test for percentage comparison and Post-hoc ANOVA for mean comparison (two tailed)

Italics: < 0.01, bolditalic: < 0.05

#### Working conditions

Table 4 presents the working conditions of the study participants. A majority of nurses worked in surgical wards (79.2%), had no management responsibility (92.3%), had a permanent contract with the hospital (80%), rated their current work as not suitable for a nursing professional (71.3%), perceived high work pressure (78.7%), and had had at least one opportunity for career training (89.7%). Half of the nurses reported that their job frequently involved care for patients with severe conditions (47%), had conflicts with coworkers (55.2%), and had normal to bad relationships with supervisors (50.3%) and patients (58%). More than 90% of participants intended to continue working at the hospital in the next 5 years.

**Table 4:** Working conditions of the whole sample and three clusters

Work conditions	Whole sample (n = 600)		Cluster						Overall		Between clusters		
			1 (n = 105)		2 (n = 133)		3 (n = 362)				1 to 2	1 to 3	2 to 3
	n	%	n	%	n	%	n	%	χ^2^	p	p	p	p
Wards													
Surgical wards	475	79.2	82	78.1	111	83.5	282	77.9	1.9	0.39	0.32	1.00	0.21
Others^b^	125	20.8	23	21.9	22	16.5	80	22.1					
Management responsibility													
Yes	46	7.7	6	5.7	28	21.1	12	3.3	43.9	*<* *0.01*	*<* *0.01*	0.26	*<* *0.01*
No	554	92.3	99	94.3	105	78.9	350	96.7					
Employment contract type													
Temporary	120	20.0	33	31.4	2	1.5	85	23.5	39.8	*<* *0.01*	*<* *0.01*	0.13	*<* *0.01*
Permanent	480	80.0	72	68.6	131	98.5	277	76.5					
Care for severe patients													
Frequently	282	47.0	60	57.1	67	50.4	155	42.8	7.5	***0.02***	0.36	***0.01***	0.15
Occasionally to none	318	53.0	45	42.9	66	49.6	207	57.2					
Extra work out of responsibilities													
Frequently	97	16.2	23	21.9	28	21.1	46	12.7	8.1	***0.02***	0.88	***0.03***	***0.03***
Occasionally to none	503	83.8	82	78.1	105	78.9	316	87.3					
Work suitable for profession													
Not suitable	428	71.3	63	60.0	91	68.4	274	75.7	10.5	*<* *0.01*	0.22	*<* *0.01*	0.11
Suitable	172	28.7	42	40.0	42	31.6	88	24.3					
Work suitable for health													
Not suitable	349	58.2	46	43.8	72	54.1	231	63.8	14.5	*<* *0.01*	0.12	*<* *0.01*	0.06
Suitable	251	41.8	59	56.2	61	45.9	131	36.2					
Work suitable with income													
Not suitable	107	17.8	12	11.4	29	21.8	66	18.2	4.4	0.11	***0.04***	0.12	0.37
Suitable	493	82.2	93	88.6	104	78.2	296	81.8					
Self-assessment of work pressure													
High	472	78.7	91	86.7	110	82.7	271	74.9	8.4	***0.02***	0.47	***0.01***	0.07
Normal to low	128	21.3	14	13.3	23	17.3	91	25.1					
Opportunity to higher training													
Never	62	10.3	18	17.1	3	2.3	41	11.3	15	*<* *0.01*	*<* *0.01*	0.13	*<* *0.01*
Yes	538	89.7	87	82.9	130	97.7	321	88.7					
Relationship with coworkers													
Normal to bad	235	39.2	67	63.8	43	32.3	125	34.5	32.6	*<* *0.01*	*<* *0.01*	*<* *0.01*	0.67
Good	365	60.8	38	36.2	90	67.7	237	65.5					
Conflict with coworkers													
Yes	331	55.2	66	62.9	75	56.4	190	52.5	3.6	0.16	0.35	0.07	0.48
Never	269	44.8	39	37.1	58	43.6	172	47.5					
Relationship with supervisor													
Normal to bad	302	50.3	77	73.3	59	44.4	166	45.9	27.1	*<* *0.01*	*<* *0.01*	*<* *0.01*	0.84
Good	298	49.7	28	26.7	74	55.6	196	54.1					
Conflict with supervisor													
Yes	108	18.0	29	27.6	36	27.1	43	11.9	23.2	*<* *0.01*	1	*<* *0.01*	*<* *0.01*
Never	492	82.0	76	72.4	97	72.9	319	88.1					
Relationship with patients/customers													
Normal to bad	348	58.0	75	71.4	69	51.9	204	56.4	10.2	*<* *0.01*	*<* *0.01*	*<* *0.01*	0.42
Good	252	42.0	30	28.6	64	48.1	158	43.6					
Intention to continue working at the hospital in the next 5 years													
Yes	567	94.5	97	92.4	129	97.0	341	94.2	2.56	0.28	0.12	0.49	0.25
No	33	5.5	8	7.6	4	3.0	21	5.8					

^a^p from Chi square test for percentage comparison (two tailed)

^b^This group included preclinical wards and voluntary health examination department, rehabilitation center

Italic: < 0.01, bolditalic: < 0.05

Working conditions varied significantly across the three clusters. Cluster 1 consisted of more nurses with: a temporary work contract (31.4%), perceived high work pressure (86.7%), and unfavorable relationships at work with coworkers (63.8%), supervisor (73.3%) and patients (71.4%). In contrast, a majority of those in cluster 2 were permanent staff (98.5%), had received training (97.7%), and had good relationships with coworkers (67.7%) and supervisors (55.6%). More cluster 2 nurses participated in career training and had management responsibility than did those in clusters 1 and 3. Cluster 3 had distinct features with higher percentages of nurses regarding their work as not suitable for a nursing professional (75.7%) and their work as not suitable for their current health condition (63.8%). No significant differences were found between the 3 clusters in ward type, conflicts with coworkers and intention to continue working in the hospital in the next 5 years.

Clusters 1 and 2 shared several similarities in terms of frequently caring for patients with severe conditions, extra work, perception of work as not suitable for a nursing professional, high work pressure, and conflicts with coworkers and supervisors in comparison with cluster 3. Clusters 1 and 3 had similar conditions of work, with smaller percentages of nurses having management responsibility, a temporary work contract, and less opportunity of training than cluster 2. There were significant differences between cluster 2 and 3 in employment contract type, management responsibility, extra work, training opportunities and conflicts with supervisors (most at p < 0.01).

## Discussion

The aim of this study was to investigate stress, anxiety and depression in nurses working in a major surgical hospital in Hanoi, and to explore the heterogeneity among clinical nurses in terms of demographic characteristics and working conditions. The findings indicate three broad clusters among study participants, which differ from each other in prevalence and severity of indicators of single and multi-mental disorders, and in demographic characteristics and working conditions.

The findings concerning self—reported stress, anxiety and depression in this study were inconsistent with previous findings. The prevalence of anxiety in this study was congruent with results from previous studies among nurses in developing countries, but the prevalence of stress and depression were considerably lower [6, 25, 33, 36, 51]. Despite this, the prevalence of mental health problems among clinical nurses was still greater than that of the general population or other healthcare practitioners [52]. Not many studies have focused on anxiety [29], particularly among nurses. However, the high prevalence of anxiety found clearly implies the need for further research on this area. As far as we are aware, this is the first study to determine the combination of concurrent psychological problems among nurses. These findings emphasize the importance of targeting different, co-existing psychological dimensions in epidemiological and intervention studies.

The characteristics that defined cluster 1 warrant particular attention, since nurses in this cluster reported high prevalence of single mental health problem and a combination of mental health problems. Clearly identification of factors contributing to both high rates and co-occurrence of multiple mental health problems in this group would provide better evidence to inform the development of sound and cost-effective prevention and intervention programs. Participants in this cluster were in the transition phase of career development when they were assigned more complex tasks suitable for their experience (high demand) but without managerial authority, and therefore limited job control. Additionally, imbalanced demand-reward (high work pressure but less opportunity for career training and low income) [53], emotional burden of caring for patients with more severe conditions [16], responsibility for extra administrative work [18] are all known to produce greater psychological strain. Moreover, work conflicts and poor relationships were also more common among nurses in this cluster. Conflicts with supervisors may be expected to reduce job resources and support which can increase mental strain [54]. Nursing is service work that requires a variety of effective interpersonal interactions. High work pressure and caring for patients with more severe conditions requires nurses to have more contacts with physicians, other nurses and patients’ relatives with whom they may have conflicted relations, amplifying their mental distress [55].

Exploring the particular characteristics of cluster 2 would be helpful in identifying psychological buffer or resilience factors in stressful hospital working environments. Although nurses in this cluster shared some similarities with participants in cluster 1, the prevalence of self-reported mental health problems in cluster 2 was significantly lower and less severe than that of cluster 1. Older age, permanent employment, and longer years of working in the hospital helped nurses to gain more work and life experience, more opportunities for career development such as training and promotion, increased job security and higher levels of satisfaction [19, 40, 56]. Moreover, nurses in this group were not only stable at work but also in their private lives with more than 90% of participants in a stable marital relationship. Having a spouse in a relationship plays an important role in emotional stability and lowers the risk of psychiatric morbidity, particularly for depressive symptoms [6, 20, 21]. However, the percentage of self-perceived mental disorders in cluster 2 were larger than that of cluster 3, possibly because of higher prevalence of chronic diseases in this group. Better general health has been reported as a protective factors for better mental health [6, 21, 23].

In contrast to participants in clusters 1 and 2, cluster 3 nurses reported the lowest prevalence of mental health problems. A majority of nurses in this groups were younger and had better physical health (low prevalence of chronic diseases). Results from this study are inconsistent with previous research which has reported younger age as a predictor of depression [20]. In this Vietnamese hospital, younger nurses were less exposed to and responsible for caring for patients with severe conditions in comparison with more senior nurses in the other two clusters. Their lack of training opportunities and management responsibilities were expected and understandable since relatively newly recruited nurses usually receive hands-on instruction from senior nurses with long years of services, and with recognized contribution to the work of the hospital being a requirement for career promotion. Due to their lack of experience, nurses in cluster 3 were more likely to be assigned physical tasks which posed less psychological challenge than the nursing professional tasks of patient care [57]. These conditions also created less conflicts between younger nurses in this cluster and their older counterparts, which might explained the lower percentage of bad relationships or conflicts at work among nurses in cluster 3.

This study was limited to nurses and did not include other occupational groups at the same hospital. While including other occupational groups was outside the scope of this study it would be valuable to study other occupational groups in order to clarify factors that may be unique to nurses or to other occupational groups and to identify commonalities across occupational groups.

### Limitations of the study

The study was conducted in only one tertiary hospital specialized in surgery. Therefore the generalization of results might be limited to nurses working in similar settings, such as intensive care units, emergency rooms, or surgical wards. However, this study’s results may be applicable to hospitals at provincial and higher level that are characterized by heavy workloads and high task demands.

The cross-sectional study design of the study identifies only associations and no causal inferences may be made. Nor can a cross-sectional study identify factors that contribute to an individual moving from one cluster to another over time. These matters can only be investigated in longitudinal follow-up studies.

The use of self-administered questionnaires admits the possibility of bias [58]. Under psychological stress, participants may either under- or over-report details of working conditions or negative perceptions [23]. Future studies should, if possible, employ other more objective methods data collection, such as direct observation of working conditions. More robust study designs, such as cohort or longitudinal studies, are more suitable.

We acknowledge that personality factors and exposure to other adverse events are likely to contribute to stress, anxiety and depression, and that these factors were not explicitly measured in this study.

Although the strength of cluster analysis lies in its ability to generate meaningful sub-groups in data, it also has several problems. The choice of variables strongly influences the characteristics of the generated sub-groups. No clear theoretical underpinning is available to guide the selection of variables for the classification of subjects. An additional problem is the lack of reliability checks to assess the fit of cluster solutions [27].

Despite these limitations, this study is the first study to attempt to study the co-occurrence of stress, anxiety and depression in an occupational group. The results of the study emphasize the importance and advantages of investigating multiple mental dimensions concurrently in order to better understand the reality of workplace mental health.

## Conclusions

Nearly half of clinical nurses suffered from at least one mental problem and 7.3% reported all three conditions—stress, anxiety and depression. The prevalence of self-perceived stress, anxiety and depression were 18.5%, 39.8% and 13.2% respectively. Managerial role, opportunities for career development, marital relationship, longer years of working in the hospital, better physical health status, and harmonious work relationship and environment were associated with lower rates and severity of self-reported mental disorders.

The findings of high levels of mental health problems, and of the co-occurrence of multiple mental health problems, among nurses have some clear implications for mental health policy and for hospital management. They point to the urgent need to develop workplace mental health policies, workplace mental health promotion programs and effective supports within workplaces for workers experiencing significant mental health problems. These developments are essential for improving quality of services and safety of patients and staff in high-pressure environments such as hospitals. They are also essential for improving productivity in such organizations.

## Additional file

**Additional file 1.** Questionnaires on demographic data and working conditions.

## Abbreviations

DASS: Depression, Anxiety and Stress scale
