# Supplementary material for: Stress, anxiety and depression in clinical nurses in Vietnam: a cross-sectional survey and cluster analysis
Source: Int J Ment Health Syst. 2019 Jan 3;13:3. doi: 10.1186/s13033-018-0257-4 (PMC6317201; doi:10.1186/s13033-018-0257-4)
Supplement: Supplementary file 1 — Additional file 1. Questionnaires on demographic data and working conditions. [file 13033_2018_257_MOESM1_ESM.docx]

Appendix: Questionnaires on demographic data and working conditions

| HANOI UNIVERSITY  OF PUBLIC HEALTH |  |
| --- | --- |

| Code: |  |  |  |
| --- | --- | --- | --- |

**SELF ADMINISTRATION QUESTIONNAIRES**

**FACTOR RELATED TO OCCUPATIONAL STRESS, ANXIETY AND DEPRESSION**

**Introduction:**

Good morning, I am a lecturer from Hanoi University of Public Health. I collect information on stress, anxiety and depression and related factors among nurses in order to provide evidence for future interventions in in the hospital. This research is conducted in collaboration between Hanoi University of Public Health and your hospital.

Would you please provide some personal information and opinions regarding this topic. The information you provide will be used only for the purpose of this research.

If you agree to join this research, please provide following information:

Date:……../……./…………….

Name (not compulsory): …………………………………………….

Email (write your email if you want to receive results of this research): ......................

**Instructions:**

Please circle the number (code) appropriate with your answer or directly write your answer in the blank space.

| **No** | **Questions** | **Answers** | **Code** | **Instruction** |
| --- | --- | --- | --- | --- |
| A | **Demographic information** | | | |
| A1 | Which year were you born? | ………………… |  | Write the year |
| A2 | What is your gender? | Male | 1 | *Circle 1 appropriate answer* |
|  |  | Female | 2 |  |
| A3 | What is your highest level of education? | High school | 1 | *Circle 1 appropriate answer* |
|  |  | Pre-intermediate Vocational training | 2 |  |
|  |  | Intermediate Vocational training | 3 |  |
|  |  | College | 4 |  |
|  |  | University (undergraduate) | 5 |  |
|  |  | University (post graduate) | 6 |  |
| A4 | Are you married or in relationship? | Single and live with family | 1 | *Circle 1 appropriate answer* |
|  |  | Single and live alone | 2 |  |
|  |  | Married | 3 |  |
|  |  | Separate | 4 |  |
|  |  | Divorced/ widow/ widower | 5 |  |
| A5 | How much pressure of family income/ finance do you have? | In charge of all family finance | 1 | *Circle 1 appropriate answer* |
|  |  | 50% | 2 |  |
|  |  | A small part | 3 |  |
|  |  | Almost no pressure | 4 |  |
| A6 | At the moment, do you suffer from following diseases? | Compensated occupational disease and have social insurance for that disease | 1 | *Circle all appropriate answers* |
|  |  | Metabolism disorders | 2 |  |
|  |  | Musculoskeletal disorders | 3 |  |
|  |  | Cardiovascular disorders | 4 |  |
| A7 | When did you start working in this hospital? | Month/year:……………..  (example: July 2008) |  | *Write appropriate month and year* |
| B | **Working conditions** | | | |
| B1 | Which ward are you working in this hospital? | Ward name: …….. |  | *Write the name of the ward you are currently working in* |
| B2 | Are you in charge of management responsibility? | No | 1 | *Circle 1 appropriate answer or write your answer* |
|  |  | Yes, I am ward head nurse | 2 |  |
|  |  | Yes, other management responsibility (please specify………  ……………………………………) | 3 |  |
| B3 | What type of work contract do you have with this hospital? | Temporary, over 6 months | 1 | *Circle 1 appropriate answer* |
|  |  | Temporary, no time limit | 2 |  |
|  |  | Permanent contract | 3 |  |
|  |  | Other, specify………………. | 4 |  |
| B4 | Do you have to care for severe/ dying patients? | Frequently (1 - 2 times/week) | 1 | *Circle 1 appropriate answer* |
|  |  | Sometimes (1 - 2 times/ month) | 2 |  |
|  |  | Yes but few (1-2 times per year) | 3 |  |
|  |  | No, never | 4 |  |
| B5 | Do you have to do tasks out of responsibility? | Very frequently | 1 | *Any tasks assigned/ required by superior or coworkers, not your personal choice* |
|  |  | Frequently | 2 |  |
|  |  | Occasionally | 3 |  |
|  |  | No, never | 4 |  |
| B6 | Is your current job suitable for your nursing professional? | Very unsuitable | 1 | *Circle 1 appropriate answer* |
|  |  | Unsuitable | 2 |  |
|  |  | Normal | 3 |  |
|  |  | Suitable | 4 |  |
|  |  | Very suitable | 5 |  |
| B7 | Is your current job suitable for your health condition? | Very unsuitable | 1 | *Circle 1 appropriate answer* |
|  |  | Unsuitable | 2 |  |
|  |  | Normal | 3 |  |
|  |  | Suitable | 4 |  |
|  |  | Very suitable | 5 |  |
| B8 | Is your current job suitable for your level of income? | Very unsuitable | 1 | *Circle 1 appropriate answer* |
|  |  | Unsuitable | 2 |  |
|  |  | Normal | 3 |  |
|  |  | Suitable | 4 |  |
|  |  | Very suitable | 5 |  |
| B9 | How do you evaluate your current work pressure? | Very high | 1 | *Circle 1 appropriate answer* |
|  |  | High | 2 |  |
|  |  | Normal | 3 |  |
|  |  | Low | 4 |  |
|  |  | Very low | 5 |  |
| B10 | Do you have opportunity to attend career training? | No, never | 1 | *Circle 1 appropriate answer* |
|  |  | No, but I will have in the next few years | 2 |  |
|  |  | I had attended training once or twice | 3 |  |
|  |  | I attend one or two training courses every year | 4 |  |
| B11 | How do you evaluate your relationship with coworkers? | Not good/ Bad | 1 | *Circle 1 appropriate answer* |
|  |  | Normal | 2 |  |
|  |  | Good | 3 |  |
| B12 | Have you ever had conflict with coworkers? | Yes, frequently | 1 | *Circle 1 appropriate answer* |
|  |  | Yes, sometimes | 2 |  |
|  |  | No, never | 3 |  |
| B13 | How do you evaluate your relationship with your superior? | Not good/ Bad | 1 | *Circle 1 appropriate answer* |
|  |  | Normal | 2 |  |
|  |  | Good | 3 |  |
| B14 | Have you ever had conflict with your superior? | Yes, frequently | 1 | *Circle 1 appropriate answer* |
|  |  | Yes, sometimes | 2 |  |
|  |  | No, never | 3 |  |
| B15 | How do you evaluate your relationship with your patients and their family? | Not good/ Bad | 1 | *Circle 1 appropriate answer* |
|  |  | Normal | 2 |  |
|  |  | Good | 3 |  |
| B16 | If your working conditions are unchanged, do you intent to leave this hospital in the next 5 years? | Yes | 1 | *Circle 1 appropriate answer* |
|  |  | No | 2 |  |
